# Novel Therapeutic Strategies for Malignant Salivary Gland Tumors: Lessons Learned from Breast Cancer

**DOI:** 10.1155/2011/187623

**Published:** 2011-11-21

**Authors:** Ryuichi Murase, Tomoki Sumida, Akiko Ishikawa, Rumi Murase, Sean D. McAllister, Hiroyuki Hamakawa, Pierre-Yves Desprez

**Affiliations:** ^1^Department of Oral and Maxillofacial Surgery, Ehime University Graduate School of Medicine, Ehime 791-0295, Japan; ^2^California Pacific Medical Center Research Institute, San Francisco, CA 94107, USA

## Abstract

Malignant salivary gland tumors (MSGTs) account for 2–6% of all head and neck cancers. Despite the rarity, MSGTs have been of great interest due to a wide variety of pathological features and high metastasis rates resulting in poor prognosis. Surgical resection followed by radiation therapy represents the main treatment of this malignancy. Adjuvant therapy is reserved for the management of local recurrence, no longer amenable to additional local therapy, and for metastasis. Based on the studies from other types of tumors, particularly breast cancer, the expression and function of sex steroid hormone receptors in cancer have been extensively studied and applied to diagnosis and treatment. Although a number of studies in MSGTs have been published, the rationale for hormone therapy is still controversial due to the disparate results and insufficient number of cases. However, some recent reports have demonstrated that certain salivary gland neoplasms are similar to breast cancer, not only in terms of the pathological features, but also at the molecular level. Here, we shed light on the biological similarity between MSGTs and certain types of breast cancer, and describe the potential use of hormone and additional therapies for MSGTs.

## 1. The Role of Sex Steroid Hormone Receptors in Cancer Therapy

The function of sex steroid hormone receptors in breast cancer has been extensively studied and applied to diagnosis and treatment [1, 2]. Estrogen stimulates cell proliferation of breast epithelial cells, and the close relationship between the expression of estrogen receptor (ER) and the prognosis of breast cancer has been well characterized [3]. Progesterone levels fluctuate during the menstrual cycle and regulate cell proliferation and differentiation; however, less is known regarding its role in breast cancer [4–6]. We have previously identified that introducing progesterone receptor (PR) into hormone-independent breast cancer cells significantly suppressed their proliferative and invasive activities upon progesterone treatment [7].

Several drugs such as Tamoxifen, an estrogen receptor antagonist, as well as a synthetic progestin similar to progesterone, are considered to be effective at inhibiting tumor cell proliferation. These drugs are given as adjuvant therapies in breast cancer patients when greater than 10% of the breast cancer cells express ER or PR receptors as assessed by immunohistochemical staining of the tumor tissue [8, 9]. Molecular targeted drug therapy is generally defined as less toxic than traditional chemotherapy; however, some studies have reported severe side effects, and carefully designed and regulated clinical trials are necessary to confirm the safety. Moreover, these types of therapies are not viable when a tumor expresses a low level of a molecular target, for example, a receptor [10]. This problem is exemplified in breast cancers which do not express ER, PR, or HER2 receptors, that is, triple negative cases. In this patient population, it is a challenge for clinicians to provide efficacious treatments. 

Sex steroid hormone therapy in prostate cancers is based on their high sensitivity to androgen inhibition. The most common hormone therapy is initiated by reducing the concentration of circulating androgens through surgical or medical castration and/or by administering antiandrogens such as flutamide or bicalutamide [11, 12]. However, in almost all patients, the efficacy of the treatment decreases over time as the tumor becomes “androgen refractory” [13]. As a result, these patients develop distant metastases, such as in bone, which eventually is fatal to the patient. The molecular events, therefore, which control the transition from androgen-sensitive prostate cancer to androgen-refractory prostate cancer need to be elucidated.

Accumulating evidence suggests that the androgen receptor (AR) plays a critical role in regulating the growth of both androgen-sensitive and androgen-refractory prostate cancer [14–19]. In addition, recent studies showed that the AR can regulate invasion and metastasis [20]. In AR-negative cell lines such as PC3 and DU145, it has been shown that forced AR expression decreased invasive properties and treatment with androgen further reduced invasion of these cells [21, 22]. Moreover, it was reported that hormone refractory prostate cancers demonstrated a variety of AR alterations that were either not found in hormone naïve tumors or found at lower frequency [19]. A more recent investigation has demonstrated that forced expression of AR, in a subline of a metastatic androgen-dependent prostate cancer cell line, led to increased invasion [20]. It is clear that a more detailed understanding of the AR alterations in the evolution of androgen-refractory prostate cancer is needed to help drive the development of potential new therapies.

Regarding the potential applications of hormone therapies against other cancers, some studies in ovarian and colon cancer have been described [23]. In ovarian cancer, the use of estrogen as a menopausal therapy has frequently been associated with an increased risk of ovarian cancer, and there is still conflicting evidence on the impact hormone therapy has on decreasing the risk of cancer [24]. A recent study, however, suggests that this problem can be circumvented by coadministering progestin and estrogen [25]. Further, experiments in culture showed that progesterone reduced proliferation of both benign and malignant ovarian tumor cells [26]. Therefore, progestin might be a key factor to prevent and suppress ovarian cancer cell growth. In contrast to ovarian cancer, estrogen appears to have protective effects against colon cancer [27]. However, the role of hormone replacement therapy involving estrogen for treatment of colon cancer is poorly understood, and further analyses are needed.

## 2. Pathological and Biological Similarities between MSGTs and Breast Cancer

Mammary and salivary glands are tubuloacinar exocrine glands and share similar morphological characteristics. Comparing the tumors arising from these two different sites, similar histological features are observed [28–31]. Although the cancers differ in incidence and clinical behavior, certain biological features identified in both entities have been described, and potential common therapeutic approaches have been considered. 

The WHO classification of MSGTs represents more than twenty different histological subtypes [32, 33]. The majority of them are divided into two groups, the secretory duct origin (including mucoepidermoid carcinoma (MEC) and salivary duct carcinoma (SDC)) and the intercalated origin type (including adenoid cystic carcinoma (ACC)) [34, 35]. Most of these tumors occur in the parotid gland (70%), and less than 25% are malignant [36]. Although the incidence of tumors at other sites, such as submandibular, sublingual, and minor salivary glands, is less common, the proportion of malignancy is higher, approximating 50% [36]. Most of aggressive breast cancers are composed of invasive ductal carcinoma, and other histologic features such as MEC and ACC are relatively rare. Below, we briefly describe some of the types of MSGTs that display common features (at the morphological and molecular levels) with breast cancers and, therefore, could provide potential common therapeutic strategies.

### 2.1. Mucoepidermoid Carcinoma (MEC)

MEC is the most common salivary gland neoplasm, accounting for 29–34% of all malignancies of the major and minor salivary glands [32]. These tumors grow slowly and present painless massed in most cases. They are primarily composed of intermediate, mucous, and epidermoid cells. The cell types are classified histologically as low, intermediate, and high grade, and 5-year overall survival (OS) varies from 92% to 100% in low-grade tumors, 62% to 92% in intermediate-grade tumors, and 0% to 43% in high-grade tumors [37]. High-grade MEC is an aggressive malignancy, characterized by high rate of local recurrence and distant metastasis. On the contrary, low-grade MEC generally do not metastasize. 

 On the other hand, MEC of breast is a rare entity with an estimated incidence of 0.2% and is composed of a mixture of basaloid, intermediate, epidermoid, and mucinous cells [30, 38]. Since Patchefsky et al. first described breast MEC in 1979, only 28 cases have been reported [38–53]. Because of the rarity, the prognosis is still controversial. However, it was described that MECs deriving from breast and salivary glands share similar biological features and morphologies [30]. They classified breast MECs into three grades using the same grading system as for the salivary gland tumors and demonstrated that high-grade tumors show high mortality resulting from lymph node and distant metastasis. These results suggest that MECs from both mammary and salivary glands, having similar morphological features, could have similar treatment strategies. 

Further, a common cytogenetic alteration of breast and salivary MECs has been reported. A reciprocal translocation t(11;19)(q21;p13) (MAML2:MECT) was shown in breast MEC, which is known as the most frequent genetic alteration of salivary gland counterparts [54]. This translocation creates a fusion product (MAML2:MECT1) that activates transcription of cAMP/CREB target genes [54, 55]. Another report described that the expression of the protein fusion gene was associated with a significantly lower risk of death compared to those without the fusion protein MAML2:MECT1 [56]. It has also been shown that other subtypes of breast cancers are negative for this gene, suggesting that this fusion gene is specific to MEC. This translocation is likely to be a promising marker for MECs from both breast and salivary gland [57].

### 2.2. Adenoid Cystic Carcinoma (ACC)

ACCs account for 22% of MSGTs [58]. There are three histological subtypes: tubular, cribriform, and solid [29, 59]. In contrast to squamous cell carcinomas that account for the vast majority of head and neck malignancies, ACC often spreads systemically, especially to lung and bone, and the metastatic proportion of this type of neoplasm represents 24–55% [60]. Due to the high metastatic rate, prognosis is poor. The 10-year OS is 39–55%, and 20-year OS is 21–25% [60].

**Figure 1:**
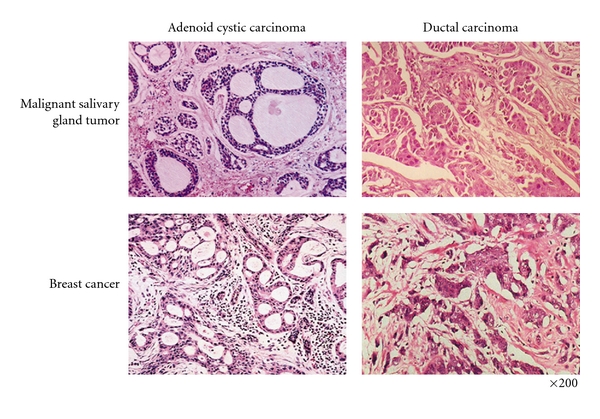
*Histological comparison of malignant salivary and mammary gland tumors*. Salivary glands and mammary glands are both tubuloacinar exocrine tissues sharing similar morphological features. It is, therefore, expected that the tumors originating from these two different glands would show similarities in their response to hormonal treatment.

On the other hand, breast ACC is a rare malignancy, accounting for 0.1–1% of all breast cancers [31]. In addition, these neoplasms show different clinical behaviors than their salivary gland counterparts. 10-year OS represents more than 90%, and lymph node and distant metastases are generally rare [31]. However, the histological features of breast ACCs are very similar to ACCs originating from salivary glands (as shown in Figure 1). Ro et al. applied the same grading system to ACCs from both types of tissues, and breast and salivary gland tumors are characterized by expression of c-KIT and share a common chromosomal translocation t(6;9) leading to the fusion gene MYB-NFIB [31, 61, 62]. c-KIT has been shown to be expressed in 80–100% of ACCs of salivary gland and in almost all ACCs from the breast [63–70]. The genetic alteration t(6;9)(q22-23;p23-24) was first identified as a characteristic for salivary gland ACCs [71]. Since then, the same translocation has been detected in breast tumors [62]. The fusion gene is highly expressed in proliferating cells and is downregulated, as the cells become more differentiated. Therefore, this gene may provide new therapeutic approaches for the management of ACCs.

### 2.3. Salivary Duct Carcinoma (SDC)

SDC is a rare and highly aggressive neoplasm representing close histologic features with invasive ductal carcinoma of the breast (IDC) [28, 72, 73]. SDC generally shows more aggressive features and less survival rates than other MSGTs. The epithelium tends to form cribriform, papillary, and solid growth patterns along with the duct-like structures [28]. The morphology of SDC is characterized by cuboidal and polygonal cells forming distended ducts and solid nests (often with central necrosis) and which are very similar to comedo carcinomas of the breast [28]. In addition to the histopathological resemblance, both entities have similar clinical behaviors; that is, they have highly metastatic features leading to poor prognosis.

A wide variety of molecular studies have been performed and led to the identification of certain biological markers for SDCs. Among them is HER-2, which is amplified in 20–25% of breast cancers [74, 75]. Various studies of HER-2 in SDC have shown variable results, with amplification ranging between 25% and 100% of the tumors [76]. Nonetheless, the proportion is much higher than in other histological subtypes such as ACCs and MECs described above [8, 28, 32, 36, 77–84]. HER-2 expression is considered to correlate with histological grades in both salivary gland neoplasms as well as breast cancer, and it represents a potential attractive therapeutic approach for SDCs. As HER-2 can also enhance AR function, antiandrogen therapy may be effective against MSGTs when HER-2 is overexpressed.

Previous studies have described that high EGFR expression in SDCs may contribute to tumor growth [78, 85]. EGFR has been shown to enhance tumorigenesis in several human carcinomas by blocking apoptosis and promoting angiogenesis [86]. Regarding both EGFR and HER-2, the interaction with hormonal pathways has also been described. In the breast and uterus cancers, treatment with anti-EGF antibodies reduced tumor proliferation induced by treatment with estradiol. Likewise, the antiestrogen ICI 164,384 reduced the effects of EGF-induced tumor proliferation [87]. 

Hoang et al. performed molecular studies, using microsatellite markers and DNA flow cytometry, and compared the biological characteristics underlying SDC and IDC. They determined that there were similar allelic alterations on chromosomal arms 6q, 16q, 17p and 17q, and that DNA aneuploidy in both malignancies may contribute to their aggressive behavior [88]. Recently, it was determined that polysomy of chromosome 7 was detected in 25% of SDCs and this alteration correlated with poor OS [89]. This correlation was also reported in IDCs and supported the notion that EGFR gene mutations may guide therapy [90]. Taken together, gene alterations of both EGFR and HER-2 may define molecular features of these two types of malignancies, and these receptors may be candidates for targeted therapy.

## 3. Hormone Therapy for the Treatment of Patients with MSGTs

As described above, several types of MSGTs are morphologically and biologically similar to malignant breast cancers [29, 91] (Figure 1). Further, the clinical significance of sex hormone receptors has been debated since White and Garcelon first described therapy with estrogen against salivary gland neoplasms in 1955 [92]. Previous reports obtained using a low number of biopsy samples have shown conflicting results regarding the expression of sex hormone receptors, making difficult to determine the potential benefits of hormone therapy [93–103]. Therefore, additional studies are required in order to clarify the role of hormone receptors in MSGTs.

Although several studies examined ER and PR expression in MSGTs, there is substantial disparity in the results: the expression of ER and PR can vary between 0–86% and 0–50%, respectively [93–103]. This disparity may be explained by differences in antibodies used, experimental methods for detection (e.g., Western blotting versus immunohistochemistry), and the criteria used to rule out false positives and negatives. It is, therefore, particularly critical to standardize protocols (such as for the IHC) similar to that described for the analysis of breast cancer tissues. In addition, some of the differences might result from insufficient number of samples available. 

Even though ER expression is unlikely to represent a useful marker for detecting MSGTs, a subset of MSGTs clearly expresses hormone receptors, and these receptors could control disease progression. Thus, current therapeutic strategies used in breast cancer patients may also be effective for the treatment of MSGTs. Moreover, the feasibility of hormone therapy seems to be supported by the accumulating reports of AR expression in SDCs. Although the expression of AR is generally rare in salivary gland neoplasms, SDCs commonly express AR in 92–100% of the cases [85, 104, 105]. Recently, Jaspers et al. reported that androgen deprivation therapy (ADT) in patients with recurrent or disseminated disease showed a clinical benefit in five out of ten cases, and two of them had partial responses [76]. This approach is, therefore, more effective than the results obtained after the use of chemotherapy. Given the fact that ADT has generally less adverse effects than chemotherapy, antiandrogen therapy may lead to better clinical outcome and become a standard treatment method for SDCs.

Williams et al. described that most tumors derived from breast and salivary glands expressed estrogen receptor-beta (ER-*β*) and that the patients whose tumors lacked ER-*β* were at higher risk for local recurrence [8]. In addition, previous studies have linked the loss of ER expression to aggressive features in adenocarcinomas from breast, prostate, and colon [106–111]. In breast and prostate carcinoma, ER-*β* has been shown to inhibit cell proliferation by cyclin D1 pathway and to induce apoptosis by downregulating bcl-2 and/or inducing Bax expression [112, 113]. Targeting ER-*β* may, therefore, become a useful approach for the management of salivary duct carcinoma.

**Figure 2:**
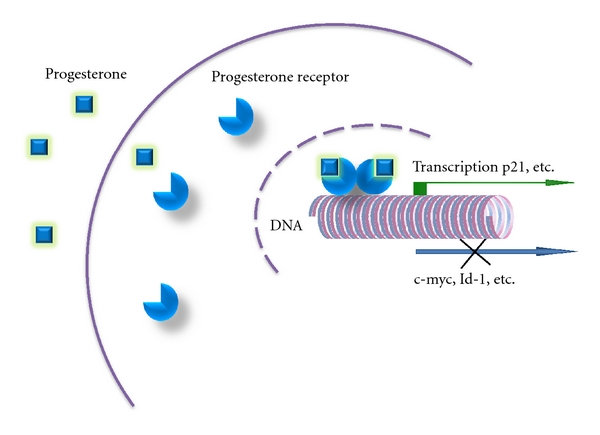
*Pg suppresses proliferation and invasion of both salivary gland and breast cancer cells.* In our recent studies, the inhibitory effect of Pg on the proliferative and invasive activities of the salivary gland and breast tumor cells was demonstrated, suggesting some common mechanisms. In both types of cancers, expression of Id-1 and c-myc was downregulated after Pg treatment, whereas p21 expression level was upregulated.

In our previous studies, we determined that MSGTs cell lines in culture lacked estrogen and progesterone receptors. However, lack of hormone receptors may be a consequence of malignant transformation and may represent a requirement for the establishment of immortal cell lines. Studies have reported the efficacy of Tamoxifen against MSGTs in patients [114, 115], and one of them showed a long-term survival even though ER was not detected by immunohistochemistry. This result appears to be supported by another case report, where Tamoxifen could reactivate ER expression [116]. Our previous studies showed that progesterone could suppress MGST cell aggressiveness similarly to that observed in breast cancer cells (Figure 2). Specifically, we demonstrated that after transduction of PR, progesterone could significantly suppress proliferation (and invasion) of MSGT cells [117]. This suppression did not lead to cell death but to cell-cycle arrest. These data suggest that if MSGTs express significant levels of PR, then progesterone treatment may slow the growth of the primary tumor and potentially shift it to a dormant state. Since most MSGTs occur in older age patients, triggering tumor dormancy could improve quality of life and may be a successful way to allow the patient a normal lifespan.

Although 5-year OS in patients with MSGTs represents the average, extended survival rates are extremely low [118–120]. MSGTs show low sensitivity to chemotherapy and surgery because of anatomical limitations [121, 122]. Since radiation is also less effective, novel therapeutic approaches are eagerly anticipated. Triggering tumor dormancy as a consequence of hormone therapy would represent a novel strategy for the treatment of patients with MSGTs.

## 4. Conclusions

Besides surgical resection and radiation of MSGTs, there are no other effective therapies. Adjuvant therapy is generally reserved for palliative treatment; however, there is no clear evidence that such treatment can bring clinical benefits. Since adverse effects caused by chemotherapy often threaten the life of a patient, and since some patients with specific MSGTs, especially ACCs, show long survival even with multiple metastases, the adoption of adjuvant therapy should be carefully considered. To achieve new therapeutic methods, it is now necessary to clarify several unanswered questions such as the expression and/or function of sex steroid hormone receptors in MSGTs. As indicated by AR expression in SDCs, there is now evidence linking hormone receptors and growth factor receptors to the disease. Expression of these receptors could render the tumors sensitive to hormone therapy. However, to improve clinical outcome with rather rare malignancies, more accurate data obtained from multiple and larger studies are required. MSGTs tend to occur in older age patients, and triggering tumor dormancy could be a successful way to slow down their disease progression, therefore providing an improvement in their quality of life. Our studies in PR-negative cells also suggest that the induction of hormone receptor gene expression might be an option for delaying disease progression. Based on multiple lines of evidence from a range of cancers, sex steroid hormone receptors may prove to be appropriate targets for the establishment of novel treatments for patients with MSGTs.
